# Acceptance of deceased donor livers in the United Kingdom – development of a liver “donor utilisation index”

**DOI:** 10.3389/ti.2026.16484

**Published:** 2026-07-10

**Authors:** Jennifer Mehew, Helen Thomas, Peter Friend, Simon Knight

**Affiliations:** 1 Statistics and Clinical Research, NHS Blood and Transplant, Bristol, United Kingdom; 2 Department of Surgical Sciences, University of Oxford Nuffield, Oxford, United Kingdom; 3 Oxford Transplant Centre, Oxford, United Kingdom

**Keywords:** donation after brain death, donation after circulatory death, liver transplantation, organ donation, organ utilisation

## Abstract

Published Donor Risk Indices are often used to estimate the risk associated with transplanting a donor liver. Such indices have been constructed by considering only livers that are transplanted and estimating the risk of graft loss or patient death. Until now, there has been no index available that reflects the chance of an offered organ not being transplanted in the United Kingdom (UK). This paper presents a UK Donor Utilisation Index (UKDUI) which quantifies the impact of donor factors upon liver non-use. By comparing our developed UKDUI with a commonly used UK Donor Risk Index, we are able to see that there are differences between the factors that influence liver non-use and the factors that influence liver post-transplant outcomes. Importantly, this UKDUI can be used in future studies to characterise and identify offered livers that are either more or less likely to be transplanted. For example, the UKDUI can be used for study inclusion criteria or for risk adjustment/stratification.

## Graphical Abstract

**Figure:**
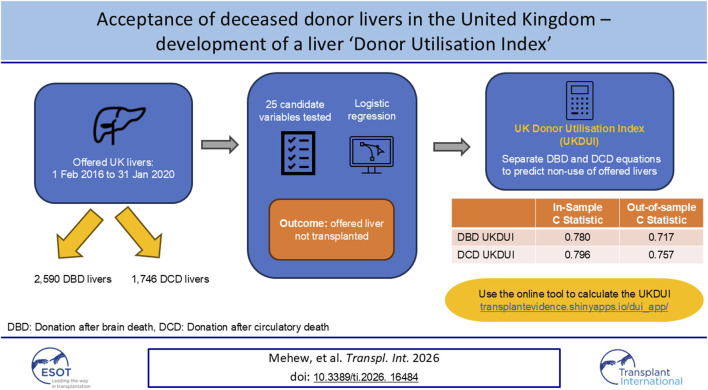

## Introduction

The dominant challenge in current-day clinical organ transplantation is the shortage of deceased donor organs. Although the number of UK deceased organ donors increased from 1,282 in the 2014/15 financial year to 1,510 in 2023/24 [1], there remains a major discrepancy between demand and supply. For instance, on 1 April 2023, there were 815 patients on the UK liver transplant list and a further 1,148 added over the course of the next 12 months; only 45% (884) were transplanted within the same 12-month period [1]. Waiting lists are even longer in other parts of the world: in the USA, the equivalent figure was 39% (9,516) for the 10,538 patients on the USA liver transplant list on 1 January 2023 and the further 13,954 patients added over the next 12 months [2].

As part of an effort to reduce the waiting time for patients in need of transplants, donor livers being offered for transplant are progressively more marginal, in terms of hypoxic injury, obesity, cardiovascular disease and age. Because of this, only 57% (854/1510) of deceased donors (where at least one organ was retrieved) in the UK resulted in a UK liver transplant in 2023/24 [1].

Until now, the degree of marginality, or risk, of offered livers in the UK has commonly been characterised using the UK Donor Liver Index (UKDLI) [3] or the US Donor Risk Index (USDRI) [4]. Such indices are useful in estimating the risk associated with transplanting such organs as these were derived from statistical models for graft survival and graft failure, respectively. However, the factors considered by these indices are therefore associated with the outcome of transplanted organs and, therefore, by definition, declined or retrieved not transplanted livers would not have been included in the model development.

It is valuable to be able to measure trends in organ utilisation over time. In order to do this accurately, changes in the degree of marginality need to be accounted for. Using the UKDLI or USDRI to characterise marginality of offered organs is not appropriate because these only apply to transplanted organs; the risk profile of offered organs is inherently different to that of transplanted organs. This study therefore focuses on non-utilisation of all livers offered for transplant following donor family consent. We aimed to assess the factors associated with liver non-utilisation (i.e., livers not transplanted) in the UK and subsequently to produce a UK liver Donor Utilisation Index (UKDUI) which can be used formally to characterise perceived marginality at the time of offer, as the risk of non-use for offered livers. If an organ has not been transplanted, the outcome had it been transplanted (i.e., the true level of marginality) will always be unknown but surgeons have chosen not to transplant it based on the perception that it would not lead to successful transplant outcomes, i.e., perceived marginality. The ability to quantify the risk of non-utilisation will be valuable in clinical studies in which utilisation is a key endpoint.

## Materials and methods

### UK liver offering scheme

In the UK, there is a separate liver offering process for DBD donors and DCD donors. On 20 March 2018, NHS Blood and Transplant (NHSBT) introduced a new named-patient National Liver Offering Scheme (NLOS) for DBD donors. The new DBD scheme uses a Transplant Benefit Score [5] for patients on the elective waiting list which helps to place the organ with the patient most likely to benefit from it. Table 1 outlines the key components of the UK liver offering scheme for adult DBD and DCD donors, including these changes.

**TABLE 1:** Description of the UK liver offering scheme.

DCD donors	DBD donors	
	Pre-20 March 2018	From 20 March 2018
1. Zonal centre offer (donor hospitals are assigned to one zonal transplant centre). Centre chooses the patient to transplant 2. Regional centre offers (each zonal centre has a set of linked centres, described as regional centres). Centre chooses the patient to transplant 3. Fast-track offers. Centre chooses the patient to transplant	1. Super-urgent patients (those who will not live long without a transplant) ordered by waiting time 2. Hepatoblastoma patients ordered by waiting time 3. Intestinal patients ordered by waiting time 4. Liver and cardiothoracic patients ordered by waiting time 5. Split liver – if donor meets the split liver criteria, a separate set of rules are followed 4. Zonal centre offer for elective patients (donor hospitals are assigned to one zonal transplant centre). Centre chooses the patient to transplant 5. Centre-based offers for elective patients. The order of the centres follows a rota. Centre chooses the patient to transplant 6. Fast-track offers. Centre chooses the patient to transplant	1. Super-urgent patients (those who will not live long without a transplant) ordered by waiting time 2. Hepatoblastoma patients ordered by waiting time 3. Intestinal patients ordered by waiting time 4. Liver and cardiothoracic patients ordered by waiting time 5. Split liver – if donor meets the split liver criteria, a separate set of rules are followed 6. Elective liver patients via either the transplant benefit score or time on the list 7. Fast-track offers. Centre chooses the patient to transplant

Further information is available at https://www.odt.nhs.uk/odt-structures-and-standards/odt-hub-programme/national-liver-offering-scheme/ and https://nhsbtdbe.blob.core.windows.net/umbraco-assets-corp/37390/pol196.pdf.

### Study population

Data from UK adult (aged ≥16 years) deceased donors where the liver was offered through the UK liver offering scheme between 1 February 2016 and 31 January 2020 were obtained from the UK Transplant Registry (UKTR) held by NHSBT. No restrictions were applied to the offer type; super-urgent, elective patient, hepatoblastoma, multi-organ, split liver, fast-track offers were all included. Donation after circulatory death (DCD) donors where the reason for non-use was recorded as ‘prolonged time to asystole’ were excluded from analysis as organ donation is not considered viable in these cases. Donors where all liver offers were withdrawn were also excluded.

Donors where the liver was transplanted outside of the UK were also excluded as we intended to assess non-utilisation of livers offered through the UK liver offering scheme. Donors where the liver was transplanted by the Dublin transplant centre were included in the analysis because “super-urgent” (Table 1) patients registered at Dublin are formally part of the national liver offering scheme. However, all other donors where the liver was transplanted in Ireland were excluded. HIV positive donors were excluded because liver utilisation for this cohort of donors is rare (no HIV donor livers (0/9) were transplanted in our cohort); the effect of HIV status upon non-utilisation would far outweigh the effect of any other donor factor if this was included in the model. Hepatitis B status was determined using Hepatitis B core antibody. Hepatitis C status was determined using both antibody and Blood Borne Virus Nucleic Acid Test (BBVNAT) testing.

Donation after brain death (DBD) donors and DCD donors were considered separately in two distinct cohorts, as the features leading to acceptance and utilisation were believed to be different. A random selection of donors representing approximately 70% of each cohort were selected for model development and 30% for model validation (Table 2).

**TABLE 2:** Number of donors used in model development and validation stages.

No. DBD donors			No. DCD donors		
Total	Model development	Model validation	Total	Model development	Model validation
3,657	2,590	1,067	2,504	1,746	758

### Statistical analysis

Non-utilisation was defined as no liver transplant resulting from a donor where the liver (whole or split) was offered for transplantation. Although the donor cohort included livers that met the UK criteria for splitting, we counted each donor only once irrespective of whether the liver was actually split. Twenty-five candidate variables collected in the UKTR were considered: 8 continuous and 17 binary/categorical (Table 3). Only variables that were known at time of offer were considered so that data missingness was not dependent upon the outcome of the offered organ; factors that were dependent on organ retrieval (e.g., steatosis) therefore could not be included. Two multivariable logistic regression models were developed to test and quantify the association of such variables with liver ‘non-utilisation’; one for DBD donors and one for DCD donors. A stepwise variable selection method was used where candidate explanatory variables were retained in the model if these reduced the model deviance significantly (p < 0.1) according to the likelihood ratio test. All explanatory variables retained in the final model were therefore statistically significant at the 10% level in the presence of other factors.

**TABLE 3:** Donor factors associated with the probability of non-utilisation with corresponding p values and odds ratios.

​	​	DBD model			DCD model		
Factor	Categorisation	P-value	Odds ratio (for non-utilisation) Higher values = less likely to be transplanted	95% CI	P-value	Odds ratio (for non-utilisation) Higher values = less likely to be transplanted	95% CI
Body Mass index (BMI)	Non-linear spline (DBD) Linear^1^ (DCD)	p < 0.0001	Figure 1	-	p < 0.0001	Figure 3	-
Alanine transaminase, ALT (iu/L)	Non-linear spline	p < 0.0001	Figure 2	-	0.01	1.001	(1.0–1.001)
Hepatitis C status	Negative	p < 0.0001	1	-	p < 0.0001	1	-
	Positive		25.16	(12.15–52.10)		26.34	(3.39–204.96)
History of alcohol abuse	None/rarely/light	p < 0.0001	1	-	p < 0.0001	1	-
	Moderate		1.37	(1.02–1.85)		1.29	(0.91–1.81)
	Heavy/Very heavy		3.08	(2.31–4.10)		2.82	(1.95–4.08)
	Stopped		0.72	(0.27–1.88)		0.72	(0.30–1.71)
Bilirubin (µmol/L)	Linear^1^	p < 0.0001	1.04	(1.03–1.06)	0.12	​	​
Age (years)	Linear^1^ (DBD) Non-linear spline (DCD)	p < 0.0001	1.03	(1.02–1.04)	p < 0.0001	Figure 4	-
Alkaline phosphate (iu/L)	Linear^1^	p < 0.0001	1.005	(1.003–1.007)	0.0004	1.005	(1.002–1.008)
History of diabetes mellitus	No	p < 0.0001	1	-	0.07	1	-
	Yes		2.14	(1.53–2.98)		1.51	(0.95–2.41)
Blood group	O	p < 0.0001	1	-	0.0003	1	-
	A		1.28	(1.02–1.62)		1.26	(0.98–1.61)
	B		1.27	(0.88–1.85)		1.33	(0.84–2.10)
	AB		3.96	(2.25–6.96)		4.55	(2.07–9.99)
Cause of death	Trauma	0.001	1	-	0.0002	1	-
	Cerebrovascular accident (CVA)		1.51	(0.73–3.10)		1.16	(0.61–2.22)
	Anoxia		0.95	(0.45–2.02)		2.16	(1.09–4.28)
	Other		2.11	(0.97–4.58)		2.69	(1.31–5.54)
Hepatitis B status	Negative	0.006	1	-	0.60	​	​
	Positive		2.23	(1.28–3.88)		​	​
History of a past tumour	No	0.02	1	-	0.04	1	-
	Yes		1.61	(1.07–2.44)		1.75	(1.00–3.05)
National liver offering scheme (NLOS) status	Liver offered before 20 March 2018	0.05	1	-	0.05	1	-
	Liver offered on or after 20 March 2018		1.24	(1.00–1.53)		1.27	(1.00–1.61)
History of cardiac or respiratory arrest	No	0.10	​	​	0.02	1	-
	Yes		​	​		0.66	(0.45–0.96)
Noradrenaline administered	No	0.12	​	​	0.33	​	​
	Yes		​	​		​	​
History of drug abuse	No	0.12	​	​	0.35	​	​
	Yes		​	​		​	​
History of cardiac disease	No	0.14	​	​	0.12	​	​
	Yes		​	​		​	​
History of hypertension	No	0.24	​	​	0.002	1	-
	Yes		​	​		1.56	(1.17–2.06)
Sodium (mmol/L)	Linear^1^	0.41	​	​	0.08	1.02	(1.00–1.04)
ITU stay prior to death (hours)	Linear^1^ (DBD) Non-linear spline (DCD)	0.44	​	​	0.0001	Figure 5	-
History of smoking	No	0.51	​	​	0.02	1	-
	Yes		​	​		1.35	(1.05–1.75)
Creatinine (µmol/)	Linear^1^	0.67	​	​	0.02	1.002	(1.0–1.004)
Sex	Male	0.85	​	​	0.02	1.37	(1.06–1.78)
	Female		​	​		1	-
Ethnicity	White	0.89	​	​	0.32	​	​
	Asian		​	​		​	​
	Black		​	​		​	​
	Other		​	​		​	​
Cytomegalovirus status (CMV)	Negative	0.91	​	​	0.36	​	​
	Positive		​	​		​	​
C statistic		0.780			0.796		

Factors found to be non-significant and hence not included in the final models are shaded in grey.

1 Also tested as a non-linear term but found not to be significant.

CI, Confidence Interval.

Missing values for any of the 25 candidate variables (Supplementary Material 2) were imputed by Multiple Imputation using the fully conditional specification method. Further details on the missing data imputation methodology are outlined in Supplementary Material 2.

All eight continuous variables were tested once as a linear variable (e.g., difference in odds of “non-utilisation,” between age 25 and 26 is the same as between age 65 and 66) and once as a non-linear variable (e.g., difference in odds of ‘non-utilisation’, between age 25 and 26 is not the same as between age 65 and 66). Natural cubic splines were used to explore non-linearity, enabling cubic expressions between “knots” at the 5%, 35%, 65% and 95% percentile values. A model containing the spline terms was considered more appropriate than a model containing just the linear term if it reduced the model deviance significantly (p < 0.1) according to the likelihood ratio test. Interaction terms were not considered in the interest of parsimony and to reduce the risk of over-fitting.

Separate DBD and DCD liver Donor Utilisation Index (UKDUI) equations were derived from the linear predictor of the resulting two models. As the models were developed from logistic regression, the UKDUI value was equivalent to the ‘probability of non-utilisation’ whereby only values between 0 and 1 were possible, with higher values representing donors perceived to be more marginal and hence higher risk of non-utilisation. Providing the calibration (or predictive ability) of the DBD model was comparable with that of the DCD model, UKDUI values from the DBD model could be compared with UKDUI values from the DCD model.

Rigorous model checking and validation was performed for both the DBD and DCD models. The methodology used is outlined in Supplementary Material 1.

All analyses were performed using SAS version 9.4 (SAS Institute Inc., NC, USA).

### Using the UKDUI

To illustrate how the UKDUI equation can be used, the UKDUI was calculated for a range of hypothetical donors with differing characteristics. The UKDLI was also calculated for these donors for reference. To understand how the UKDUI compares with the UKDLI, the UKDUI and UKDLI was calculated for each donor in the validation cohort and plotted as a scatter plot. Where there were missing data in the validation cohort for any of the UKDUI or UKDLI factors, the most common values identified from the model development cohort were used. To understand how the UKDUI and UKDLI compares in terms of ability to distinguish between livers that are transplanted and those that are not transplanted, a boxplot was created for donors in the validation dataset separately for livers that were transplanted and for livers that were not transplanted. This boxplot was performed once for UKDLI values and once for UKDUI values, for DBD and DCD donors separately.

## Results

### Model development

**FIGURE 1:**
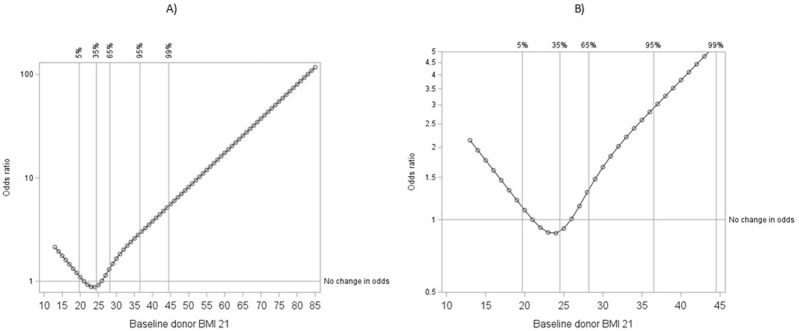
Odds ratios for Body Mass Index (BMI) relative to a BMI = 21 donor (Donation after Brain Death (DBD) model). **(A)** Full range of observed BMI values. **(B)** BMI values up to 45 (99% of observed data).

**FIGURE 2:**
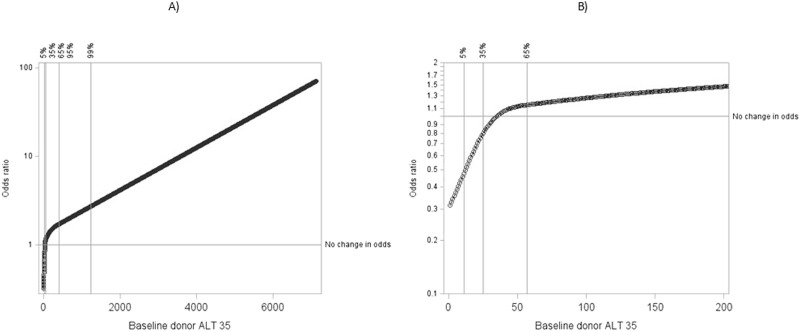
Odds ratios for Alanine transaminase (ALT) relative to an ALT = 35 donor (Donation after Brain Death (DBD) model). **(A)** Full range of observed ALT values. **(B)** ALT values up to 200 (89% of observed data).

**FIGURE 3:**
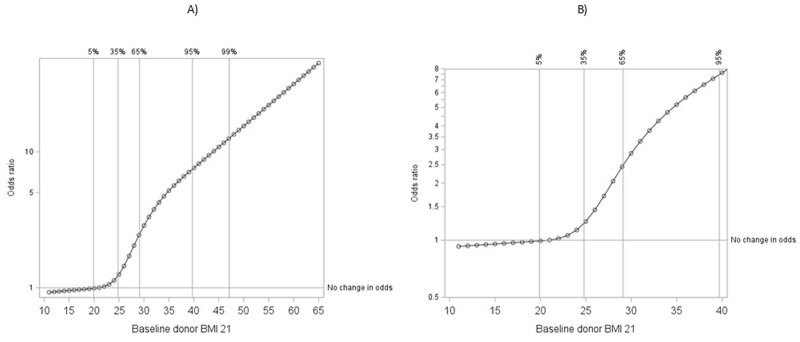
Odds ratios for Body Mass Index (BMI) relative to a BMI = 21 donor (Donation after Circulatory Death (DCD) model). **(A)** Full range of observed BMI values. **(B)** BMI values up to 40 (95% of observed data).

**FIGURE 4:**
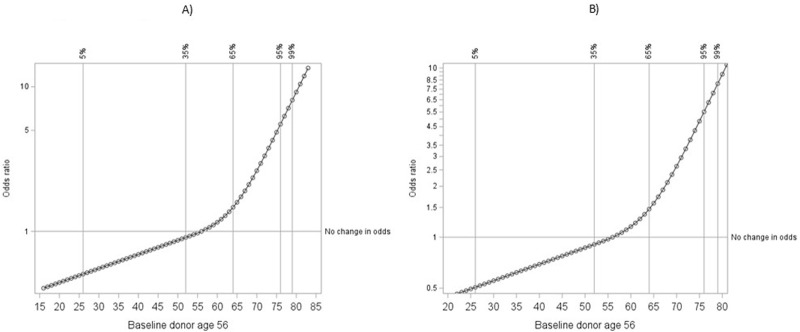
Odds ratios for age relative to an age = 56 donor (Donation after Circulatory Death (DCD) model). **(A)** Full range of observed age values. **(B)** Ages 20–80 only (97.5% of observed data).

**FIGURE 5:**
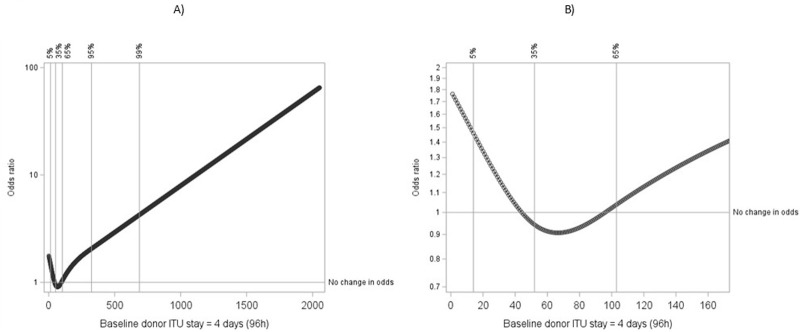
Odds ratios for Intensive Therapy Unit (ITU) Stay relative to an ITU Stay = 96 h donor (Donation after Circulatory Death (DCD) model). **(A)** Full range of observed ITU stay values. **(B)** ITU stay values up to 168 hrs (7 days) (83% of observed data).

Of the 2,590 DBD donors whose livers were offered in the development dataset, 2,015 (78%) were transplanted. This figure was lower for DCD donors with 538 out of 1,746 (31%) being transplanted. The factors found to be significantly associated with non-utilisation and associated odds ratios are shown in Table 3 for DBD and DCD donors separately. The p values for those factors not found to be significant, and hence not included in the final models, are also shown in Table 3 shaded in grey. Note that the effects of donor Body Mass Index (BMI) and alanine transaminase (ALT) upon liver non-use were found to be non-linear for DBD donors, as were the effects of BMI, age and ITU stay for DCD donors. The odds ratios for these factors are illustrated by Figures 1–5 respectively. Figure 1 shows that livers from DBD donors with a BMI around 25 were most likely to be transplanted, but the odds of non-utilisation increased 1) as BMI increased higher than 25 and 2) as BMI decreased lower than 25. This counter-intuitive effect for lower (<25) BMI donors was felt to be due to confounding factors not incorporated by the model; low BMI in donors may be associated with other medical factors. For DCD donors, Figure 3 shows that there was little effect of BMI upon non-utilisation unless the donor had a BMI greater than approximately 23 after which point the odds of non-utilisation increased as BMI increased. Figure 2 shows that higher values of ALT lead to higher odds of non-utilisation for DBD donors. The change in odds is more drastic for low ALT values; the difference between ALT values of 14 and 15 in terms of odds is greater than for ALT values of 49 and 50. Figure 4 shows that for DCD donors, the odds of non-utilisation increase as donor age increases. This effect is amplified for donors over the age of 60; the difference between age 65 and 66 in terms of odds is greater than the difference between age 40 and 41. Figure 5 shows that livers from DCD donors with an ITU stay between approximately 60–80 h were most likely to be transplanted, but the odds of non-utilisation increased 1) as ITU stay increased higher than 80 h and 2) lower than 60 h. This counter-intuitive effect for shorter ITU stays (<60 h) was felt to be due to confounding factors not incorporated by the model; shorter ITU stays may be associated with less stable donors.

The resulting UKDUI equations derived from the logistic regression models presented in Table 3 are shown in Equation 1 (for DBD donors) and Equation 2 (for DCD donors). Note that if the equations are to be used with prospectively collected data, or for livers offered on or after 20 March 2018, the ‘post NLOS’ term can be added on to the intercept term (−4.3888 + 0.2157 for DBD donors and −5.4499 + 0.2354 for DCD donors) and held fixed. Details of the UKDUI spline terms can be found in Supplementary Material 3 (DBD) and Supplementary Material 4 (DCD).

The model checking and model validation performed suggested both the DBD and DCD models were an adequate representation of liver non-utilisation (Supplementary Material 5). In particular, the in-sample C statistic (which represented the model’s ability to discriminate between utilised and non-utilised livers within the development dataset) was 0.780 for the DBD model and 0.796 for the DCD model. Furthermore, the out-of-sample C statistic (evaluating discrimination within the validation dataset) was 0.717 for the DBD model and 0.757 for the DCD model.

### Using the UKDUI

For those wishing to use the UKDUI to estimate the probability of non-utilisation for a particular donor, there is an online UKDUI calculator [6].

Table 4 illustrates the UKDUI values for various hypothetical donors. For comparison, UKDLI values are also shown for these donors. Note that UKDUI values can range from 0 to 1 but no such restriction applies to the UKDLI (values are usually between 1 and 4 although can sometimes be higher). Where missing data occurred for the donor factors included in the UKDUI or UKDLI equations, the values in Supplementary Material 6 were imputed. A description of each hypothetical donor is provided in Supplementary Material 10 along with an explanation for the similarities and disparities between calculated UKDUI and UKDLI values.

**TABLE 4:** UKDUI values for various hypothetical donors (differences from Donor A highlighted in bold).

Donor	A	B	C	D	E	F	G
**UKDUI**	**0.03**	**0.16**	**0.98**	**0.99**	**0.12**	**0.46**	**0.83**
**UKDLI**	0.90	1.69	1.75	3.31	1.39	0.90	1.69
Donor type	DBD	**DCD**	DBD	**DCD**	**DCD**	DBD	**DCD**
Hepatitis B status	Negative	Negative	**Positive**	**Positive**	Negative	Negative	Negative
Hepatitis C status	Negative	Negative	Negative	Negative	Negative	**Positive**	**Positive**
History of diabetes mellitus	No	No	**Yes**	**Yes**	No	No	No
National liver offering scheme status	Post 20 March 2018	Post 20 March 2018	Post 20 March 2018	Post 20 March 2018	Post 20 March 2018	Post 20 March 2018	Post 20 March 2018
History of a past tumour	No	No	No	No	No	No	No
Age (years)	20	20	**68**	**68**	20	20	20
Bilirubin (µmol/L)	9	9	**17**	**17**	9	9	9
Alkaline phosphate (iu/L)	75	75	**159**	**159**	75	75	75
Cause of death	Trauma	Trauma	**CVA**	**CVA**	Trauma	Trauma	Trauma
Blood group	O	O	**B**	**B**	O	O	O
History of alcohol abuse	None/rarely/light	None/rarely/light	**Heavy/very heavy**	**Heavy/very heavy**	None/rarely/light	None/rarely/light	None/rarely/light
Body Mass index (BMI)	18.5	18.5	**45**	**45**	18.5	18.5	18.5
Alanine transaminase, ALT (iu/L)	35	35	**150**	**150**	35	35	35
History of smoking	No	No	**Yes**	**Yes**	No	No	No
Sex	Male	Male	Male	Male	**Female**	Male	Male
History of hypertension	No	No	No	No	No	No	No
History of cardiac or respiratory arrest	No	No	No	No	No	No	No
Creatinine (µmol/)	75	75	**150**	**150**	75	75	75
Sodium (mmol/L)	141	141	**150**	**150**	141	141	141
ITU stay prior to death (hours)	18	18	**50**	**50**	18	18	18
Height (cm) [required for UKDLI]	160	160	160	160	160	160	160

CVA: Cerebrovascular accident.

**FIGURE 6:**
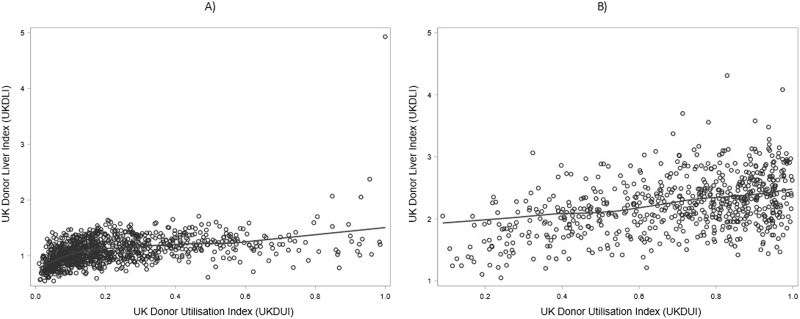
Distribution of United Kingdom liver Donor Utilisation Index (UKDUI) values in the validation dataset plotted against the corresponding United Kingdom Donor Liver Index (UKDLI) values for each **(A)** Donation after Brain Death (DBD) donor and **(B)** Donation after Circulatory Death (DCD) donor. Note that two extreme UKDLI values (UKDLI: 9.07, UKDUI: 0.84), (UKDLI:29.1, UKDUI: 0.25) have been removed from the DCD plot in order to visualise the trends more easily. Line represents a loess smoother with smoothing parameter set to 0.5.

Figure 6 illustrates the correlation between UKDUI and UKDLI values for all donors in the validation cohort. For DBD donors (Figure 6A), there appears to be some positive correlation for donor livers with UKDUI values between 0 and 0.2 suggesting that the UKDUI and UKDLI are generally in agreement for livers that are likely to be transplanted. However, there are large discrepancies between the UKDUI values and UKDLI values for livers less likely to be transplanted; the UKDUI may predict a very high chance of non-use while the UKDLI may be relatively low. For DCD donors (Figure 6B), there is some positive correlation across all values of the UKDUI however there are still large discrepancies.

**FIGURE 7:**
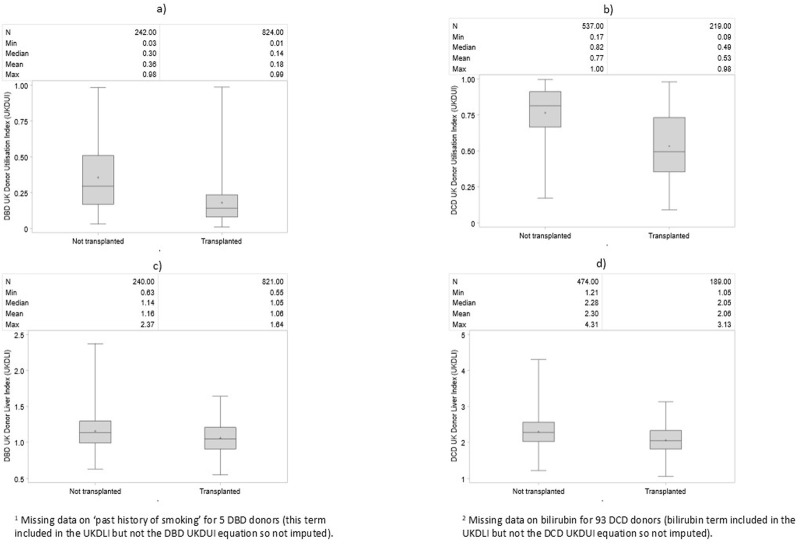
Distribution of United Kingdom liver Utilisation Index (UKDUI) values for DBD donors **(A)** and DCD donors **(B)**, and United Kingdom Donor Liver Index (UKDLI) values for DBD donors **(C)** and DCD donors **(D)**. Values are calculated from the validation dataset and split by whether the liver was transplanted or not. Note that one extreme Donation after Brain Death (DBD) UKDLI value (UKDLI: 4.93, UKDUI:1.00, liver not transplanted) and two extreme Donation after Circulatory Death (DCD) UKDLI values (UKDLI: 9.07, UKDUI: 0.84, liver not transplanted), (UKDLI:29.1, UKDUI: 0.25, liver not transplanted) have been removed from the plot in order to visualise the trends more easily.

Figures 7A,C shows that for DBD donors, the UKDUI can distinguish between livers that were transplanted from those that were not transplanted much better than the UKDLI. The same applies to DCD donor livers (Figures 7B,D).

## Discussion

Our analysis has highlighted the substantial difference in utilisation between offered UK DBD donor livers and offered UK DCD donor livers. The distribution of UKDUI values for DBD donors in the validation cohort (Supplementary Material 7A) shows that the large majority (971/1,067, 91%) of donors fell in the lower half of possible UKDUI values (UKDUI<0.5) representing lower risk of non-use whereas for DCD donors the large majority (586/758, 77%) of donors in the validation cohort (Supplementary Material 7B) fell in the upper half of possible UKDUI values (UKDUI>=0.5), reflecting the higher risk of non-use.

The strengths and weaknesses of our UKDUI are summarised in Table 5 and discussed below in further detail.

**TABLE 5:** Strengths and weaknesses of the developed UKDUI.

Strengths	Weaknesses
Can be used to assess the risk of non-use for offered organs. Previously, only indices based on the outcome of transplanted organs were available, however the risk of non-use and risk of poor outcomes post-transplant are not equivalent	Developed using UK data from 1 February 2016 to 31 January 2020 when use of machine perfusion and Hepatitis C donors was less common
Only data at time of offering is required to calculate the UKDUI, enabling the risk of non-use to be characterised for any organ at time of offer. Can be useful as entry criteria for studies that aim to target the most poorly utilised livers	Data/risk factors from further down the offering pathway are not included; e.g., long cold ischemia times or liver diagnoses upon retrieval would influence non-use but are not accounted for
Can be used to characterise liver non-utilisation across the UK as a whole. Centres that have particularly high or low non-utilisation rates, relative to the national average, can therefore be identified	Individual estimates of non-use for a particular donor do not account for variation associated with a particular centre or with an individual clinical decision-maker
Comprehensive statistical methods (including investigation of non-linear relationships) and model validation techniques have been used to produce and validate the UKDUI equations	UKDUI equations are complex and there are separate DBD and DCD equations but users are encouraged to use the UKDUI online tool [6]

The developed DBD model consisted of 13 factors found to be significantly associated with liver non-use while the DCD model had 19 factors. Despite the substantial difference between the two donor types, there were still 11 factors that were found to be significantly associated with liver non-use irrespective of donor type. Hepatitis C status was by far the most influential factor for both models as few Hepatitis C donor livers were transplanted (11/62 DBD and 1/32 DCD). However, it is acknowledged that the UK landscape has since changed such that livers from these donors are now considered for transplantation more often following the British Viral Hepatitis Group position statement [7]. As a result, the UKDUI will likely overestimate the probability of non-utilisation for Hepatitis C positive donors when considering more recent cohorts. As the effect of all other factors are considered to be independent of each other, this would have minimal impact on the applicability of the rest of the UKDUI.

Blood group AB and heavy/very heavy alcohol use was also found to be significantly associated with non-use for both DBD and DCD donors. The effect of blood group upon non-utilisation may be a result of the pool of available recipients on the list. For example, only 2% of patients on the active liver transplant list on 31 March 2024 were blood group AB [1]. The effect of sex may also be a result of other donor factors that are not easily defined/captured but are more dominant in either males or females.

Table 3 suggests that changes to national liver offering scheme made on 20 March 2018 are associated with a borderline significant increase in both DBD and DCD non-utilisation. However, the authors suspect that this simple binary indicator (pre-change, post-change) may reflect changes in the overall donor pool (over and above the donor factors considered in the modelling process) over time and an increase in offering more marginal organs, rather than the effect of the offering scheme.

The main driver for carrying out this study was the need to characterise risk of non-use when considering a UK national cohort of offered deceased donor livers. Up until now, indices based on transplanted livers have been used (UKDLI). We have shown that for both DBD and DCD donors, the UKDUI is generally better than the UKDLI at distinguishing between livers that are transplanted and those that are not (Figure 7). There have been studies outside of the UK which have aimed to characterise and predict the risk of non-use [8-16]; these are described and contrasted with the UKDUI in Table 6. Note that much of the existing literature focusses on the utilisation of retrieved organs as opposed to offered organs.

**TABLE 6:** Studies from outside the UK that aim to characterise and predict the risk of non-use for non-UK organs.

Organ	Name of index	Comparison with UKDUI
Liver (US)	Liver discard risk index (USDSRI)	A liver discard risk index (USDSRI) [8] was published in 2018 with the aim to predict the chances of liver non-use in the US. The major advantage of this index was the concept of grading livers according to the USDSRI at time of offer and preferentially offering those with higher chances of non-use to US transplant centres with a more ‘aggressive’ approach to organ acceptance. Although this is not the designed purpose of the UKDUI, it could be considered for similar organ offering concepts in the UK. The USDSRI and UKDUI were both developed using logistic regression and there is a lot of agreement between the two indices in terms of which factors influence non-use (e.g., donor age, sex, BMI, ALT, Hepatitis C status, Hepatitis B status, cause of death, bilirubin) despite representing two very different organ offering systems (US versus UK). One major difference is that the USDSRI represents both DBD and DCD donors in one equation; the effect of donor type is incorporated through one value that shifts the estimate up or down by a specified amount. The UKDUI, on the other hand, acknowledges that the factors that influence liver non-utilisation in the UK, as well as the effect of these factors, differ between DBD and DCD donors. The USDSRI also treats all factors as categorical variables whereas the UKDUI has a mixture of categorical and continuous variables Another important difference is that the USDSRI includes donors that underwent procurement surgery only
Liver (Italy)	Donor Rejected organ pre-transplantation (DROP) score	A donor Rejected organ pre-transplantation (DROP) score [9] was published in 2022 which aimed to predict the risk of liver non-use for DBD donor organs offered in Italy. Importantly, the index predicts non-use due to liver-related reasons only. The following (donor) factors as well as a term representing a nuance of the organ offering scheme were included in the index, many of which are also included in the UKDUI; age, weight, height, diabetes, Hepatitis C status, Hepatitis B status, hypotension, creatinine, ALT, AST, bilirubin Liver-related reasons for non-use were defined as; pre-procurement liver blood tests and/or imaging; gross anatomy; procurement histology, and poor perfusion. Liver-unrelated reasons for graft discard were defined as; donor tumours, donor infections, and pre-procurement donor cardiac arrest. Similarly to the UKDUI, a logistic regression model was used but for the DROP score, only liver-related reasons were treated as an event; liver-unrelated reasons were classed as a non-event along with livers that were transplanted. The logistic regression model was therefore considered a cause-specific logistic regression model under a competing risks scenario Similarly to the UKDUI, only donor factors at time of offering were considered as risk factors
Pancreas (Eurotransplant)	Pre-procurement pancreas suitability score (P-PASS)	The pre-procurement pancreas suitability score (P-PASS) was published in 2008 [10] and aimed to identify ‘suitable’ offered pancreas donors. The score consists of donor factors only; patient age, BMI, the length of stay in the intensive care unit (ICU), the occurrence of cardiac arrest, and serum levels of sodium, amylase, lipase, adrenaline, and dopamine Unlike the UKDUI, statistical modelling was not used to produce the score; the assignment of weights and points was based on medical expertise and literature review. Due to the time of its development, the P-PASS was developed primarily for DBD offered livers; no account for DCD donation was made Logistic regression was used to assess the relationship between P-PASS and the odds of accepting an offered pancreas. Using pancreas acceptance as the outcome variable, this analysis suggested that donors with a P-PASS score of ≥17 were three times more likely to be declined
Kidney (US)	US kidney donor utilisation index (USKDUI)	In 2019 a kidney donor utilisation index (USKDUI) [11] was published with the aim to predict the chances of non-use for procured kidneys in the US. Similarly to the UKDUI, a logistic regression model was developed and the following factors were found to be significant upon kidney non-use; age, hypertension, diabetes, classification as public Health service increased risk, creatinine, Hepatitis C, Stroke cause of death, BMI, donation after cardiac death, Black race, drug use (nonintravenous), current smoker, cancer, kidney donor profile index The USKDUI was then used at the centre level by multiplying each of the parameter estimates by the proportion of donor kidneys at each centre with each characteristic DBD donors and DCD donors were included in the same model but a binary factor was incorporated to represent the difference in non-use by donor type Unlike the UKDUI, this index was applicable to procured organs as opposed to offered organs
Lung (US)	Predictors of older donor lung utilisation	In 2020 a study was published [12] which identified the factors associated with the utilisation of lungs from ‘older’ solid organ donors in the US. Older donors were considered as aged >55 years. Both DBD and DCD donors were included in the same model. While not used to produce an index directly, logistic regression was used to identify the following factors associated with utilisation; sex, age, BMI, ethnicity, cigarette use, cocaine use, alcohol abuse, hypertension, diabetes, donor type, cause of death, lung function (PaO2/FiO2) ratio
Liver and kidney (US)	Use of machine learning for predicting kidney and liver discard	In 2023, a study was published [13] which aimed to predict organ utilisation/discard for livers and kidneys separately and compared six different machine learning techniques; random forest, XGBboost, FCNN, Naive Bayes, logistic regression with Lasso, ridge logistic regression (logistic regression was considered a machine learning technique). The two best performing methods in terms of predictive ability were found to be XGBoost and random forest. Interpretability is limited with some of these traditional machine learning methods and an index is not producable but if prediction is theprimary purpose then machine learning is worth consideration
Kidney (US)	Using machine learning to identify factors, additional to the existing kidney donor risk index, that influence kidney discard	A study was published in 2025 [14] which emphasised the importance of having an interpretable and easy-to-implement model for the prediction of non-use of retrieved kidneys The model included all factors included in the existing US kidney donor risk index (USKDRI) [15] as a minimal set of variables and then additional factors were added 1) through random forest machine learning and 2) logistic regression. Cold ischemia time and warm ischemia time were included as factors meaning that this model could not be used at time of offer or be used to predict offered organs that were not retrieved. Models were developed both with and without biopsy information For the model excluding biopsy information, in addition to the KDRI variables, the following factors were found to be influential upon kidney non-use: creatinine, age, hypertension, arginine, BMI, hisotry of smoking, donor type, coronary angiogram, height
Kidney (US)	Probability of discard or delay (PODD)	The probability of discard or delay (PODD) was published in 2010 [16] for US kidneys from donors where at least one kidney had been retrieved. This index aimed to identify factors that influenced whether a kidney would be 1) discarded (retrieved not transplanted) or delayed (cold ischemia time >36 h) or 2) transplanted Logistic regression was used to model this outcome variable. As two kidneys were studied from most donors (leading to non-independence), donor‐level clustering was accounted for using a clustered sandwich estimator of the variance–covariance matrix The following (donor) factors were included in the model; sex, age, blood group, BMI, cancer, history of smoking, hypertension, myocardial infarction history, diabetes, insulin treatment, donor type, cause of death, human T lymphocyte virus, cytomegalovirus, Hepatitis B, Hepatitis C, CDC high risk donor, pumped, sclerosis, creatinine DBD and DCD donors were included in the same model/equation and the authors investigated different cutoffs for PODD values in order to predict a kidney that was ‘easy to place’ or ‘hard to place’

Only donor data available at the time of offering were included. This enabled us to produce a UKDUI that characterises perceived marginality at the time of offer and moreover, include data that were recorded consistently for livers that were 1) offered not retrieved, 2) retrieved not transplanted and 3) transplanted. In practice, further organ/donor information may have come to light during or after retrieval, for example, causing the transplanting surgeon to decline the organ; this is not captured in the UKDUI. As a result, there will be systematic variation in the data which is unaccounted for. However, the bias that would be caused by missing data for the cohort of offered not retrieved organs if we included later datapoints was considered more problematic than our chosen methodology.

Figure 6 showed there to be large discrepancies between the UKDUI and UKDLI values for livers less likely to be transplanted. There are two explanations for such discrepancy. The UKDLI is designed to describe the risk of graft failure if the organ was to be transplanted whereas the UKDUI is designed to describe the risk of not transplanting the organ. The discrepancy shown in Figure 6 could therefore suggest that transplant centres are declining some DBD livers that have a low risk of graft failure. The other explanation for such discrepancy is that the UKDUI contains more factors than the UKDLI meaning that the UKDUI is much more variable depending on donor characteristics. The UKDLI was constructed using data on transplants performed between 2000 and 2014 and perhaps if more recent data were used, the UKDUI and UKDLI could be in stronger agreement. Or perhaps the factors that influence graft survival genuinely are different (fewer) than the factors that influence liver utilisation decision making.

Future studies need to carefully consider which index is more appropriate to use. The UKDUI does not predict graft outcomes; it is designed to describe the risk of an offered liver not being transplanted. It should therefore be used in organ utilisation studies. However, the UKDLI would be more appropriate for studies looking at the graft outcome of transplanted livers. We therefore recommend that the UKDUI is used if wishing to assess the risk of non-use and that a transplant outcome-based index (such as the UKDLI) is used if wishing to assess the risk of poor transplant outcomes. There is increasing importance in using organ utilisation as both a trial endpoint and a focus for interventions. There are technologies which have shown promise in increasing utilisation (e.g., Normothermic Machine Perfusion [17, 18] and Normothermic Regional Perfusion [19]), and these technologies will be most efficient if targeted to the most poorly utilised donor organs. An accurate UKDUI allows us to do this both for inclusion criteria in the clinical trial setting, but also for efficient use in clinical practice. For instance, the UKDUI presented in this paper was used as inclusion criteria in the PLUS liver clinical trial (https://doi.org/10.1186/ISRCTN11552402). Studies in other organ areas have also started to focus on utilisation as an important endpoint, for example, the PITHIA kidney clinical trial [20]. Similar tools for these organs may help to target these interventions increasing efficiency. It should be highlighted that focussing on utilisation as a clinical endpoint alone does not give a full picture; increased utilisation is only a positive outcome if accompanied by adequate clinical outcomes.

Our developed UKDUI has many strengths; it has been developed on a large national dataset and validated in a randomised national subset which has shown the index to reflect UK liver utilisation between 1 February 2016 and 31 January 2020 well (Supplementary Material 5). The statistical models used to create the UKDUI were found to have good predictive ability (Table 3). It should be noted however that no external validation has been performed; the period following 31 January 2020 covers the COVID-19 pandemic during which time liver utilisation was substantially impacted. Furthermore, as the UKDUI was used as an inclusion criteria for recruitment of livers into the PLUS clinical trial (https://doi.org/10.1186/ISRCTN11552402) between 11 April 2022 and 3 April 2023, the liver utilisation landscape will have differed during the trial period due to increased use of machine perfusion.

Of course, attitudes towards risk and organ utilisation may differ between clinicians and between transplant centres, and this may also be affected by waiting list composition. The developed UKDUI provides a useful summary of liver non-utilisation across the UK as a whole.

Use of machine perfusion during the study period was fairly low, relative to the current UK landscape, so future work could be applied to investigate how the probability of non-utilisation changes in the presence of machine perfusion, or any other ‘new’ factor of interest, once the known risk factors incorporated in the UKDUI are adjusted for.

For those wishing to use the UKDUI but have missing data for some of the donor factors included in the UKDUI equations, it is suggested that the values presented in SDC 6 are imputed. While the UKDUI equations are complex, users are encouraged to use the UKDUI online tool [6].

In addition to providing a tool to help understand donor case mix, this UKDUI offers utility as a clinical audit tool; it will enable future studies to adjust for the risk profile (risk of non-utilisation and perceived marginality) of offered livers. In doing so, studies will be able to quantify and compare behaviour between transplant centres and even between clinicians’ individual willingness to accept organs. Risk adjustment using the UKDUI will also help to quantify trends in liver utilisation over time. Future studies may find value in validating the UKDUI in a different healthcare environment (e.g., USA or Eurotransplant).

Equation 1:

$$UK{DUI}_{DBD}=\frac{\exp\left(\eta_{DBD}\right)}{1+\exp\left(\eta_{DBD}\right)}$$ (1)

$$\eta_{DBD}=-4.3888+\left(0.8005\;if\;HepB\;positive\right)+\left(3.2254\;if\;HepC\;positive\right)+\left(0.7601\;if\;past\;history\;of\;diabetes\right)+\left(0.2157\;if\;post\;NLOS\right)+\left(0.4792\;if\;past\;history\;of\;tumour\right)+0.030043\;age+0.042627\;bilirubin+0.005034\;alk\;phos+\left(0.3155\;if\;moderate\;alcohol\;consumption\right)+\left(1.1254\;if\;heavy/very\;heavy\;alcohol\;consuption\right)-\left(0.3342\;if\;stopped\;consuming\;alcohol\right)+\left(0.2501\;if\;blood\;group\;A\right)+\left(0.2428\;if\;blood\;group\;B\right)+\left(1.3751\;if\;blood\;group\;AB\right)+\left(0.4115\;if\;cause\;of\;death\;CVA\right)-\left(0.0484\;if\;cause\;of\;death\;Anoxia\right)+\left(0.7446\;if\;cause\;of\;death\;Other\;\left(excluding\;Trauma\right)\right)+BMIspline+ALTspline$$

Post-NLOS: liver offered on or after 20 March 2018

Equation 2:

$${UKDUI}_{DCD}=\frac{\exp\left(\eta_{DCD}\right)}{1+\exp\left(\eta_{DCD}\right)}$$ (2)

$$\eta_{DCD}=-5.4499+\left(0.3026\;if\;past\;history\;of\;smoking\right)+\left(3.2712\;if\;HepC\;positive\right)+\left(0.4152\;if\;past\;history\;of\;diabetes\right)+\left(0.3163\;if\;male\right)+\left(0.2354\;if\;post\;NLOS\right)+\left(\;0.5582\;if\;past\;history\;of\;tumour\right)+\left(0.4415\;if\;past\;history\;of\;hypertension\right)-\left(0.4145\;if\;past\;history\;of\;cardiac\;or\;respiratory\;arrest\right)+0.000652\;ALT+0.016575\;sodium+0.004917\;alk\;phos+0.002071\;creatinine+\left(0.2510\;if\;moderate\;alcohol\;consumption\right)+\left(1.0362\;if\;heavy/very\;heavy\;alcohol\;consuption\right)-\left(0.3321\;if\;stopped\;consuming\;alcohol\right)+\left(0.2293\;if\;blood\;group\;A\right)+\left(0.2847\;if\;blood\;group\;B\right)+\left(1.5143\;if\;blood\;group\;AB\right)+\left(0.1481\;if\;cause\;of\;death\;CVA\right)+\left(0.7689\;if\;cause\;of\;death\;Anoxia\right)+\left(0.9891\;if\;cause\;of\;death\;Other\;\left(excluding\;Trauma\right)\right)+BMIspline+agespline+ITUspline$$

Post-NLOS: liver offered on or after 20 March 2018

## Data Availability

The data analyzed in this study is subject to the following licenses/restrictions: The analyses described in this article were carried out at NHSBT, who maintain the UK Transplant registry on behalf of transplant centers in the United Kingdom. NHSBT are reliant on the General Data Protection Regulation Article 6(1)(e) - Performance of a public task. This lawful basis requires specific exemptions under Article 9(2)(h) and also (i) and (j). This allows NHSBT to use patient identifiable information for service evaluation without the consent of patients. In order to maintain data minimisation under GDPR, data may only be released on request. Requests to access these datasets should be directed to Jennifer.Mehew@nhsbt.nhs.uk.
