# Supplementary material for: Acceptance of deceased donor livers in the United Kingdom – development of a liver “donor utilisation index”
Source: Transpl Int. 2026 Jul 10;39:16484. doi: 10.3389/ti.2026.16484 (PMC13395755; doi:10.3389/ti.2026.16484)
Supplement: Supplementary file 1 [file Supplementaryfile1.docx]

**Capsule sentence summary:**

Using a large national dataset, we have established a UK liver Donor Utilisation Index (UKDUI) which characterises the risk of non-use for livers offered for transplantation.

**SM 1: Model checking and validation methodology**

**Model Checking**

For both models, the Hosmer and Lemeshow C statistic (equivalent to the area under the ROC curve) was calculated which assesses predictive ability. A value of 0.5 indicates that the model predicts no better than adopting a 50/50 guess and a value of 1 indicates perfect prediction, whereas values above 0.7 show acceptable predictive ability. Standardised deviance residuals were assessed to identify any outlying observations. Cooks D statistic was plotted for each observation to identify any values that were highly influential in the calculation of the set of parameter estimates (referred to as influential observations). The Delta-betas were also plotted against each observation for each parameter in the model to identify any observations that highly influenced particular parameter estimates.

**Model Validation**

The UKDUI value was calculated for each donor in the DBD and DCD validation cohorts (**Table 1**) using the developed models described above. Missing values were imputed using the most common values (for categorical variables) or median values (for continuous variables) from the respective development cohorts as opposed to using Multiple Imputation because this would be the recommended imputation method for using the UKDUI in practice. For each validation cohort (DBD and DCD), donors were divided into four groups defined by the quartile UKDUI values from the respective development datasets (after imputation). A logistic regression model for the probability of non-utilisation was fitted to the validation dataset in order to assess odds ratio differences between these four groups of donors. The distribution of UKDUI values was also compared between the donors in the validation dataset whose livers were transplanted and those whose livers were not transplanted.

**SM 2: Missing Data**

**Missing Data Methodology**

Missing data were estimated from logistic regression for binary variables, a discriminant function approach for categorical variables and linear regression for continuous variables. For some variables however (DBD model variables: Hepatitis C status; Noradrenaline use, DCD model variables: Hepatitis C status; Noradrenaline use; Hepatitis B status, donor history of a past tumour), the discriminant function approach had to be used due to low numbers of events and hence lack of convergence. All potential variables which were to be tested in the modelling process, along with a ‘liver retrieved’ (Yes/No) and a ‘liver transplanted’ (Yes/No) indicator, were incorporated by these imputation methods to estimate such missing values. Twenty imputed datasets were generated and subsequent analyses were performed on the combined dataset using weights dependent upon the degree of missingness for each variable tested. Model summaries were performed using the proc MIanalyse statement in SAS which combined all 20 datasets. The distribution of values for each variable in the imputed dataset were compared to the observed distribution to check for similarity and the range of imputed values was checked for consistency between imputations. All continuous variables required log transformation to ensure non-negative estimates. Imputation was performed for the DBD and DCD model development cohorts separately (imputation was not required for the validation cohorts).

**Donor variables tested and percentage missing**

| **Factor** | **Missing values**  **N (%)** | |
| --- | --- | --- |
|  | **DBD Model Development Cohort**  **N=2,590** | **DCD Model Development Cohort**  **N=1,746** |
| Body Mass Index (BMI) | 0 (0%) | 0 (0%) |
| Alanine transaminase, ALT (iu/l) | 403 (15.6%) | 262 (15.0%) |
| Hepatitis C status | 4 (0.2%) | 4 (0.2%) |
| History of alcohol abuse | 48 (1.9%) | 40 (2.3%) |
| Bilirubin (µmol/l) | 295 (11.4%) | 180 (10.3%) |
| Age (years) | 0 (0%) | 0 (0%) |
| Alkaline phosphate (iu/l) | 272 (10.5%) | 187 (10.7%) |
| History of diabetes mellitus | 19 (0.7%) | 19 (1.1%) |
| Blood group | 0 (0%) | 0 (0%) |
| Cause of Death | 70 (2.7%) | 56 (3.2%) |
| Hepatitis B status | 4 (0.2%) | 3 (0.2%) |
| History of a past tumour | 37 (1.4%) | 37 (2.1%) |
| National Liver Offering Scheme status | 0 (0%) | 0 (0%) |
| History of cardiac or respiratory arrest | 134 (5.2%) | 89 (5.1%) |
| Noradrenaline administered | 428 (16.5%) | 196 (11.2%) |
| History of drug abuse | 57 (2.2%) | 34 (2.0%) |
| History of cardiac disease | 49 (1.9%) | 49 (2.8%) |
| History of hypertension | 43 (1.7%) | 32 (1.8%) |
| Sodium (mmol/l) | 157 (6.1%) | 101 (5.8%) |
| Intensive Therapy Unit (ITU) stay prior to death (hours) | 69 (2.7%) | 56 (3.2%) |
| Past history of smoking | 14 (0.5%) | 9 (0.5%) |
| Creatinine (µmol/) | 155 (6.0%) | 102 (5.8%) |
| Sex | 0 (0%) | 0 (0%) |
| Ethnicity | 21 (0.8%) | 23 (1.3%) |
| Cytomegalovirus status (CMV) | 14 (0.5%) | 8 (0.5%) |

DBD: Donation after brain death

DCD: Donation after circulatory death

**SM 3: Donation after Brain Death (DBD) United Kingdom liver Donor Utilisation Index (UKDUI) spline terms**

Note that (x)_+_ refers to the maximum of 0 and x.

$$BMIspline=-0.095502 BMI + 0.034105 F1\_BMI -0.062880 F2\_BMI$$

$$F1\_BMI=\frac{{(BMI-5\%)}_{+}^{3}-{(BMI-95\%)}_{+}^{3}}{95\%-5\%}-\frac{{(BMI-65\%)}_{+}^{3}-{(BMI-95\%)}_{+}^{3}}{95\%-65\%}$$

$$F2\_BMI=\frac{{(BMI-35\%)}_{+}^{3}-{(BMI-95\%)}_{+}^{3}}{95\%-35\%}-\frac{{(BMI-65\%)}_{+}^{3}-{(BMI-95\%)}_{+}^{3}}{95\%-65\%}$$

BMI 5%=19.7, BMI 35%=24.5, BMI 65%=28.2, BMI 95%=36.5

$$ALTspline=0.041311 ALT -0.007743 F1\_ALT +0.010706 F2\_ALT$$

$$F1\_ALT=\frac{{(ALT-5\%)}_{+}^{3}-{(ALT-95\%)}_{+}^{3}}{95\%-5\%}-\frac{{(ALT-65\%)}_{+}^{3}-{(ALT-95\%)}_{+}^{3}}{95\%-65\%}$$

$$F2\_ALT=\frac{{(ALT-35\%)}_{+}^{3}-{(ALT-95\%)}_{+}^{3}}{95\%-35\%}-\frac{{(ALT-65\%)}_{+}^{3}-{(ALT-95\%)}_{+}^{3}}{95\%-65\%}$$

ALT 5%=11, ALT 35%=25, ALT 65%=57, ALT 95%=396

**SM 4: Donation after Circulatory Death (DCD) United Kingdom liver Utilisation Index (UKDUI) spline terms**

$$BMIspline=0.007428 BMI + 0.029538 F1\_BMI -0.058265 F2\_BMI$$

$$F1\_BMI=\frac{{(BMI-5\%)}_{+}^{3}-{(BMI-95\%)}_{+}^{3}}{95\%-5\%}-\frac{{(BMI-65\%)}_{+}^{3}-{(BMI-95\%)}_{+}^{3}}{95\%-65\%}$$

$$F2\_BMI=\frac{{(BMI-35\%)}_{+}^{3}-{(BMI-95\%)}_{+}^{3}}{95\%-35\%}-\frac{{(BMI-65\%)}_{+}^{3}-{(BMI-95\%)}_{+}^{3}}{95\%-65\%}$$

BMI 5%=19.9, BMI 35%=24.8, BMI 65%=29.1, BMI 95%=39.7

$$agespline=0.022407 age + 0.000002 F1\_age+0.002921 F2\_age$$

$$F1\_age=\frac{{(age-5\%)}_{+}^{3}-{(age-95\%)}_{+}^{3}}{95\%-5\%}-\frac{{(age-65\%)}_{+}^{3}-{(age-95\%)}_{+}^{3}}{95\%-65\%}$$

$$F2\_age=\frac{{(age-35\%)}_{+}^{3}-{(age-95\%)}_{+}^{3}}{95\%-35\%}-\frac{{(age-65\%)}_{+}^{3}-{(age-95\%)}_{+}^{3}}{95\%-65\%}$$

Age 5%=26, Age 35%=52, Age 65%=64, Age 95%=76

$$ITUspline=-0.014439 ITU\_stay + 0.000626 F1\_ITU-0.000985 F2\_ITU$$

$$F1\_ITU=\frac{{(ITU\_stay-5\%)}_{+}^{3}-{(ITU\_stay-95\%)}_{+}^{3}}{95\%-5\%}-\frac{{(ITU\_stay-65\%)}_{+}^{3}-{(ITU\_stay-95\%)}_{+}^{3}}{95\%-65\%}$$

$$F2\_ITU=\frac{{(ITU\_stay-35\%)}_{+}^{3}-{(ITU\_stay-95\%)}_{+}^{3}}{95\%-35\%}-\frac{{(ITU\_stay-65\%)}_{+}^{3}-{(ITU\_stay-95\%)}_{+}^{3}}{95\%-65\%}$$

ITU 5%=14, ITU 35%=52, ITU 65%=103, ITU 95%=324

**SM 5: Model checking and validation results**

**Model checking**

As the Hosmer and Lemeshow C statistics were 0.780 for the DBD model and 0.796 for the DCD model, both models were considered to exhibit clinically useful predictive ability. In general, analysis of model residuals showed nothing of concern; influential values were due to extreme values or categories which had fewer observations. Deleting any of these cases would therefore not be appropriate. Increasing the cohort size would be the only way of alleviating this issue but including donors prior to 2016 was considered not representative of current clinical practice. The DCD model suffered with issues of influence when estimating the effect of Hepatitis C status upon liver non-use as only one liver had been transplanted from a Hepatitis C positive donor. It was decided that this donor should be kept in the model as Hepatitis C is a clinically important factor for liver utilisation. However, such uncertainty should be borne in mind when interpreting the estimated odds ratio of 26 (this is reflected in the wide confidence interval of 4 to 205).

**Model validation**

The UKDUI value was calculated for each donor in the DBD and DCD validation cohorts using **Equation 1** and **Equation 2**. The imputed missing values are shown in **SM 6**. The resulting distribution of UKDUI values for the validation cohorts is shown in **SM 7A** for DBD donors and **SM 7B** for DCD donors.

The donors in the validation cohorts were then separated into four DBD ‘donor groups’ and four DCD ‘donor groups’ according to their UKDUI values. The 25%, 50% and 75% UKDUI quartile values were calculated from the DBD and DCD model development cohorts (after imputation) and were used to determine which ‘donor group’ a validation donor fell in to. **SM 8**  illustrates a clear decrease in utilisation with each UKDUI ‘donor group’. A logistic regression model was then fitted for liver non-utilisation using UKDUI ‘donor group’, a four-level categorical variable, as the only factor in the model. The results shown in **SM9** indicate that UKDUI ‘donor group’ represents the increase in non-utilisation with increasing UKDUI value well. The C statistics also suggest adequate predictive ability.

**Figure 7** compares the distribution of UKDUI values between donors whose liver was transplanted and donors whose liver was not transplanted for DBD donors (**Figure 7A**) and DCD donors (**Figure 7B**) in the validation dataset. UKDUI values were generally higher for those donors whose liver was not transplanted. For both DBD and DCD donors there was a statistically significant difference between the mean UKDUI values for donors whose liver was transplanted and donors whose liver was not transplanted (p<0.0001, Satterthwaite t-test for samples with unequal variances).

**SM 6: Missing value imputation values for model validation**

| **DBD cohort** | | **DCD cohort** | |
| --- | --- | --- | --- |
| **Factor** | **Missing values imputed as** | **Factor** | **Missing values imputed as** |
| Body Mass Index (BMI) | 26.3 | Body Mass Index (BMI) | 26.8 |
| Alanine transaminase, ALT (iu/l) | 35 | Alanine transaminase, ALT (iu/l) | 45 |
| Hepatitis C status | Negative | Hepatitis C status | Negative |
| History of alcohol abuse | None/rarely/light | History of alcohol abuse | None/rarely/light |
| Bilirubin (µmol/l) | 9 |  |  |
| Age (years) | 54 | Age (years) | 58 |
| Alkaline phosphate (iu/l) | 75 | Alkaline phosphate (iu/l) | 79 |
| History of diabetes mellitus | No | History of diabetes mellitus | No |
| Blood group | O | Blood group | O |
| Cause of Death | Cerebrovascular accident (CVA) | Cause of Death | Anoxia |
| Hepatitis B status | Negative |  |  |
| History of past tumour | No | History of past tumour | No |
| National Liver Offering Scheme status | NA (date of liver offer will always be known) | National Liver Offering Scheme status | NA (date of liver offer will always be known) |
|  |  | History of cardiac or respiratory arrest | Yes |
|  |  | ITU stay prior to death (hours) | 77 |
|  |  | Sex | Male |
|  |  | History of hypertension | No |
|  |  | Sodium (mmol/l) | 141 |
|  |  | History of smoking | Yes |
|  |  | Creatinine (µmol/) | 72 |

DBD: Donation after brain death

DCD: Donation after circulatory death

**SM 7**: Figure in separate jpeg file

Figure caption:

Distribution of United Kingdom liver Donor Utilisation Index (UKDUI) values in the validation cohort

**SM 8:** Liver utilisation by ‘donor group’ (defined using UKDUI quartile values calculated from the model development cohort)

|  | **DBD validation cohort** | | | **DCD validation cohort** | | |
| --- | --- | --- | --- | --- | --- | --- |
|  | **Donor Group Criteria** | **N** | **% transplanted** | **Donor Group Criteria** | **N** | **% transplanted** |
| **Donor Group 1** | UKDUI < 0.087 | 244 | 92% | UKDUI < 0.531 | 192 | 60% |
| **Donor Group 2** | 0.087 ≤ UKDUI < 0.159 | 266 | 86% | 0.531 ≤ UKDUI < 0.737 | 163 | 31% |
| **Donor Group 3** | 0.159 ≤ UKDUI < 0.286 | 290 | 80% | 0.737 ≤ UKDUI < 0.882 | 207 | 17% |
| **Donor Group 4** | UKDUI ≥ 0.286 | 267 | 53% | UKDUI ≥ 0.882 | 196 | 9% |

**SM 9:** Logistic regression model results for the impact of ‘donor group’ (defined using UKDUI quartile values calculated from the model development cohort) upon liver non-utilisation

|  | **DBD validation cohort** | | | | **DCD validation cohort** | | | | |
| --- | --- | --- | --- | --- | --- | --- | --- | --- | --- |
|  | **Donor Group Criteria** | **p value** | **Odds Ratio**  **(for non-utilisation)**  Higher values= less likely to be transplanted | **95% CI** | **Donor Group Criteria** | **p value** | **Odds Ratio**  **(for non-utilisation)**  Higher values= less likely to be transplanted | **95% CI** |  |
| **Donor Group 1** | UKDUI < 0.087 | p<0.0001 | 1 | - | UKDUI < 0.531 | p<0.0001 | 1 | - |  |
| **Donor Group 2** | 0.087 ≤ UKDUI < 0.159 |  | 1.87 | (1.05 – 3.31) | 0.531 ≤ UKDUI < 0.737 |  | 3.28 | (2.11 – 5.09) |  |
| **Donor Group 3** | 0.159 ≤ UKDUI < 0.286 |  | 2.86 | (1.67 – 4.91) | 0.737 ≤ UKDUI < 0.882 |  | 7.09 | (4.47 – 11.25) |  |
| **Donor Group 4** | UKDUI ≥ 0.286 |  | 10.01 | (5.97 – 16.78) | UKDUI ≥ 0.882 |  | 15.73 | (8.85 – 27.95) |  |
| **C Statistic** |  | 0.717 | | |  | 0.757 | | | |

CI: Confidence Interval

**SM10:** Comparison of UKDUI and UKDLI values for hypothetical donors

Donor A can be considered a point of reference; the characteristics that differ compared to Donor A are highlighted in bold for the other donors in **Table 4**. Donors A and B have identical characteristics except that Donor A is a DBD donor and Donor B is a DCD donor. Both have a low UKDUI (0.03 and 0.16 respectively) indicating a low probability of liver non-use, i.e. these donor livers are likely to be transplanted. This is to be expected as these are young healthy donors with no concerning past medical history. Donors C and D are DBD and DCD donors respectively, with identical characteristics to each other but they are older, less healthy donors with a history of heavy alcohol intake and smoking. As expected, their UKDUI is much higher than for Donors A and B (0.98 and 0.99 respectively) and hence these livers are unlikely to be transplanted.

Donor E is a young healthy DCD donor identical to Donor B except that the donor is female as opposed to male. This changes the UKDUI from 0.16 for male Donor B to 0.12 for female Donor E. Both have a low probability of liver non-use but the female donor slightly more so. Donor sex is not a factor in the DBD UKDUI equation so has no impact on DBD UKDUI values.

Hepatitis C status has the greatest impact on both the DBD and DCD UKDUI equations. To illustrate its impact, Donors F and G are young healthy DBD and DCD donors identical to Donors A and B, respectively, with the exception that they are Hepatitis C positive. This increases the DBD UKDUI from 0.03 for Donor A to 0.46 for Donor F and the DCD UKDUI from 0.16 for Donor B to 0.83 for Donor G. For DCD donors in particular, this highlights that Hepatitis C status can cause the probability of a liver being transplanted to change from likely to unlikely.

The factors included in the UKDLI are age, donor type, past history of smoking, sex, bilirubin, split liver status and height, all of which are included in the UKDUI except for height (the UKDUI includes BMI instead) and split liver status (this would only apply to the offered livers that were transplanted). When calculating UKDLI values for our validation cohort, we omitted the split liver term of the UKDLI equation for the same reason. **Table 4** shows a trend in UKDLI values similar to the trend in UKDUI values for each of the donor comparisons with the exception of Donors F and G because Hepatitis C status is not included in the UKDLI equation. As a result, the UKDLI values appear the same as for Donors A and B respectively.
