# Supplementary figures and images for: Acceptance of deceased donor livers in the United Kingdom – development of a liver “donor utilisation index”
Source: Transpl Int. 2026 Jul 10;39:16484. doi: 10.3389/ti.2026.16484 (PMC13395755; doi:10.3389/ti.2026.16484)

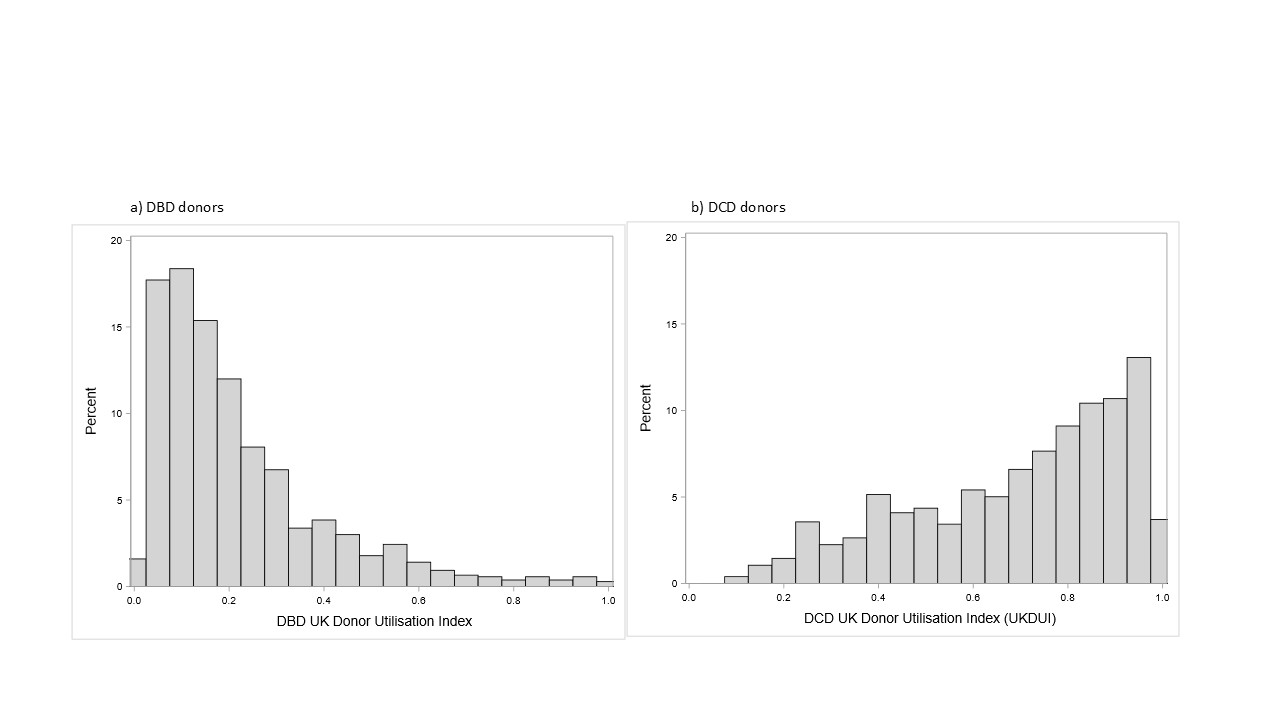

Supplement: Supplementary file 2 [file Image1.jpeg]
